# Comparison of a point-of-care serum amyloid A analyzer frequently used in equine practice with 2 turbidimetric immunoassays used in human and veterinary medicine

**DOI:** 10.1177/10406387211056029

**Published:** 2021-11-11

**Authors:** Julia Kiemle, Sarah Hindenberg, Natali Bauer, Michael Roecken

**Affiliations:** Clinic for Horses, Department of Equine Surgery and Orthopaedics, Faculty of Veterinary Medicine, Justus-Liebig-University Giessen, Giessen, Germany; Department of Veterinary Clinical Sciences, Clinical Pathology and Clinical Pathophysiology, Faculty of Veterinary Medicine, Justus-Liebig-University Giessen, Giessen, Germany; Department of Veterinary Clinical Sciences, Clinical Pathology and Clinical Pathophysiology, Faculty of Veterinary Medicine, Justus-Liebig-University Giessen, Giessen, Germany; Clinic for Horses, Department of Equine Surgery and Orthopaedics, Faculty of Veterinary Medicine, Justus-Liebig-University Giessen, Giessen, Germany

**Keywords:** horses, method validation, point-of-care assay, serum amyloid A, turbidimetric immunoassay

## Abstract

Rapid, accurate detection of serum amyloid A (SAA) is needed in equine practice. We validated a patient-side point-of-care (POC) assay (Stablelab; Zoetis) compared to the turbidimetric immunoassays LZ-SAA (TIA-Hum) and VET-SAA (TIA-Vet; both Eiken Chemical). Analytical performance was assessed at 3 different concentration ranges and with interferences. Inter-method comparison using 49 equine serum samples revealed a significant difference between median SAA results (*p* < 0.0001), with the strongest bias between the POC and TIA-Vet (median 1,093 vs. 578 mg/L). The median SAA value obtained with the TIA-Hum method was 752 mg/L. Correlation between POC/TIA-Hum and between POC/TIA-Vet was fair (r_s_ = 0.77 and 0.69) and excellent between both TIAs (r_s_ = 0.93). Bias between POC/TIA-Hum, POC/TIA-Vet, and TIA-Hum/TIA-Vet was −56.7%, –80.9%, and −28.2%, respectively. POC intra- and inter-assay CVs (16.1–30% and 19.8–35.5%) were higher than TIA CVs (generally <12%). Bilirubin and hemoglobin had a negative bias on POC and TIA-Vet results (−16.6 to −45.6%); addition of intralipid yielded a positive bias (35.9–77.4%). The POC had good linearity of SAA concentrations up to 10,312 mg/L (*R*^2^ = 0.92). A hook effect was present at SAA >3,000 mg/L for the POC assay. Equine serum SAA was stable over a median period of 2.5 y when stored at −80°C. Overall, there was excellent-to-moderate correlation between tests, but imprecision and hook effect of the POC, as well as bias between the methods, must be considered.

Serum amyloid A (SAA), the only major acute-phase protein in horses, is a highly sensitive and frequently used marker of inflammation in equine practice.^16,23,35^ Although systemic SAA in healthy horses is commonly reported as <0.5–20 mg/L,^17,26,28^ dramatic increases of up to 1,000-fold from baseline reflect inflammatory or infectious processes in diseased horses.^4,5^ In addition, its rapid increase over 6–12 h and short half-life of 30–120 min^31^ renders SAA a valuable biomarker to closely track disease severity.^7^ Different patient-side point-of-care (POC) assays^22,29,30^ and turbidimetric immunoassays (TIAs) for laboratory use^17,18^ have been validated for equine SAA measurement.

Although there is no gold standard for measurement of equine SAA,^22^ a TIA designed for measurement of human SAA concentrations (LZ-SAA, Eiken Chemical; hereafter TIA-Hum) has served as the reference method in several validation studies of equine SAA assays.^17,22,29^ Although TIA-Hum has proved reliable for equine SAA measurement,^17^ its drawback is the combination of monoclonal rat anti-human SAA1 antibody and polyclonal rabbit anti-human SAA1 antibody, which provide a high potential for batch-to-batch variation.^17,18^ Furthermore, TIA-Hum is linear only in a limited concentration range, making several dilutions and repeated analyses necessary to obtain a final SAA concentration.^17,18^

A novel TIA (VET-SAA, Eiken Chemical; hereafter TIA-Vet), designed for veterinary use and measurement of the wide SAA concentration ranges frequently encountered in horses, is available on the European market. TIA-Vet is based on the sole use of monoclonal rat anti-human SAA1 antibody, and has an acceptable reliability in measurement of extreme SAA concentrations.^18^ Contrary to TIAs, POC assays can be used stall-side, are user-friendly,^22,29^ and provide SAA results quickly and without the need for shipment of samples to a reference laboratory.^30^ To our knowledge, there are 3 POC assays available for equine SAA measurement^22,29,30^ based on the use of anti-human SAA antibody.^22^ A study describing the TIA-Hum (Eiken LZ-SAA) and POC assay (formerly Stablelab, Epona Biotech; now Stablelab, Zoetis) found the assay results unsuitable for comparison.^29^ There is no validation study comparing POC performance to the TIA-Vet assay. Furthermore, studies on the impact of interferences on POC SAA results are lacking.

Our study aims were 1) to conduct an inter-method comparison between the POC Stablelab EQ1 handheld reader with Stablelab SAA test cartridges and both previously validated TIAs (TIA-Hum and TIA-Vet) including determination of bias between methods and CVs to calculate the total observed error (TE_obs_), with the TIA-Hum assay serving as a reference method; and 2) to perform an intra-method comparison including determination of the impact of interferences, linearity, recovery, and hook effect, as well as the impact of storage.

Our hypotheses were, that 1) there is a significant bias between POC and TIA-Hum measurements and an even stronger bias between POC and TIA-Vet measurements, and 2) that equine serum SAA is stable over 2.5 y of storage at −80°C.

## Materials and methods

### Inter-method comparison

#### Study design

Study design was developed using the American Society for Veterinary Clinical Pathology (ASVCP) guidelines for quality assurance and method comparison.^8^ Our prospective study was performed March–December 2018 (validation POC assay vs. TIA-Hum) and January–February 2021 (validation POC assay vs. TIA-Vet). Our study was performed in accordance with the German Animal Welfare Act (Article 8). Ethical approval to use surplus sample volume from equine serum samples submitted for routine testing was given (Regierungspraesidium Giessen, Wetzlar, Germany, ethics committee: JLU_kTV_02_2021).

#### SAA measurement

Analyses were performed on equine serum samples submitted for SAA analyses to the Department of Veterinary Clinical Sciences, Clinical Pathology and Clinical Pathophysiology, Justus-Liebig-University Giessen, Germany. Samples were analyzed immediately (<1 h after blood collection) with the TIA-Hum test run on a clinical chemistry analyzer (Pentra 400; Horiba). The SAA results obtained during initial routine testing (subsequently TIA-Hum1) served as a reference value to assign the samples to 1 of 3 concentration ranges: SAA 0–300 mg/L (SAA_Low_), 301–1,000 mg/L (SAA_Med_), and 1,001–3,000 mg/L (SAA_High_). The 3 categories were only formed for validation purposes to assess the assay performance in different concentration ranges and do not directly correspond to the SAA RI for healthy horses, which is reported to be <20 mg/L.^35^

Samples were stored in aliquots ≥0.5 mL at −80°C until comparison with the POC assay (subsequently named POC1). TIA-Hum was performed a second time (subsequently TIA-Hum2) at the same time as POC1 and TIA-Vet results were obtained. The use of reagents, samples, and measurements of the POC assay were performed by a single trained person. The TIA assay was performed by trained laboratory technicians. If SAA concentrations exceeded the original working ranges of the TIA-Hum and TIA-Vet assays of 5–500 mg/L and 5–200 mg/L, respectively, automatic 1:6 preanalytical dilutions of 0.005 mL (TIA-Hum) and 0.002 mL (TIA-Vet) of samples were made according to the manufacturer’s instrument settings provided for the Pentra 400 analyzer. Given the extremely high SAA concentrations possible in horses, automatic sample dilution was followed by manual reflex 1:10 or 1:50 dilutions using 0.9% NaCl as required.

Internal quality control (QC) was performed once daily with QC material at 2 different concentrations for both TIA tests run on the Pentra 400 analyzer (TIA-Hum-SAA-QC-low and TIA-Hum-SAA-QC-high for TIA-Hum; VET-SAA-QC-low and VET-SAA-QC-high for TIA-Vet). Concentrations of QC material for TIA-Hum-SAA-QC-low and TIA-Hum-SAA-QC-high were 30.3 mg/L (range: 25.8–34.9 mg/L; 23.7% CV, 6.1 mg/L total bias) and 101.4 mg/L (range: 86.2–116.6 mg/L; 8.9% CV, 11.4 mg/L total bias), respectively. QC target values for VET-SAA-QC-low and VET-SAA-QC-high material were 10.2 mg/L (range: 7.7–12.8 mg/L; 8.3% CV, –1.9 mg/L total bias) and 52.3 mg/L (range: 39.2–65.4 mg/L; 7.1% CV, –6.9 mg/L total bias), respectively. Test calibration was performed using the calibration standards based on human recombinant SAA provided by the manufacturer (LZ-SAA standard Q for TIA-Hum, and VET-SAA calibrator set for TIA-Vet; Eiken Chemical). Although recommended for POC assays,^8^ POC QC was not possible because QC material was not provided by the manufacturer.

We processed 67 equine serum samples in the described fashion; 49 of 67 sera were used for determination of bias and correlation between methods. The remaining 18 of 67 sera were used for assessment of intra- and inter-assay CVs and the second part of the study, the intra-method comparison.

#### Inter-method comparison

We analyzed 49 equine serum samples spanning the concentration ranges SAA_Low_, SAA_Med_, and SAA_High_ with the POC, TIA-Hum, and TIA-Vet tests. After a median storage of 2.5 y (0.3–4.8 y) following initial TIA-Hum1 measurements during routine testing, POC1 and TIA-Hum2 measurements were conducted simultaneously in March–December 2018. TIA-Vet measurements were performed in January–February 2021 (median 2.5 y [range: 2.0–3.8 y] later, when the species-specific TIA-Vet test kits were available). In addition to the mentioned storage time difference, SAA measurements also differ by an additional freeze–thaw cycle performed after the comparison between POC1 and TIA-Hum2.

For statistical analysis, statistical software programs (v.17.8.6, MedCalc Software; Prism 6, GraphPad Software) were used. A *p*-value ≤ 0.05 was considered significant. For the inter-method comparison, POC1, TIA-Hum2, and TIA-Vet results were used. Bland–Altman analysis was performed, and both mean percent bias and mean absolute bias were calculated. Calculation of Spearman rank correlation coefficient was performed, interpreting correlations as “excellent” for Spearman rho (r_s_) = 0.93–0.99, “good” for r_s_ = 0.80–0.92, “fair” for r_s_ = 0.59–0.79, and “poor” for r_s_ < 0.59, respectively.^20,27^ Additionally, Passing–Bablok analysis was performed. Before analysis, the data were visually inspected for adequate distribution over the investigated analyte range.

#### Precision

Intra- and inter-assay precision was assessed for each of the 3 assays at 3 SAA concentrations (i.e., SAA_Low_, SAA_Med_, and SAA_High_). Intra-assay variation was determined by analyzing 10 replicates of each concentration range on the same day; for calculation of the inter-assay variation, 1 replicate was run at each test level over 10 consecutive days, using a different single aliquot thawed every day. Intra- and inter-assay CVs, SAA mean (M), and SD were calculated for POC, TIA-Hum, and TIA-Vet assays from replicate measurements.

Imprecision was calculated based on M and SD for both intra- and inter-assay results:

$$\mathrm{CV}\left[\%\right]=\frac{\mathrm{SD}}{M}\times 100\%$$

Given that no quality requirements are available on the desired CV for equine SAA assays, the observed (analytical) CV (CV_a_) was compared to quality requirements derived from biologic variation (i.e., the intra-individual CV [CV_i_] of 25% as described for human SAA measurement).^21^ Quality requirements were fulfilled if:

$${\mathrm{CV}}_{a}\left[\%\right]<0.5\times {\mathrm{CV}}_{i}^{34};\;\;i.e.,\;\;{\mathrm{CV}}_{a}<12.5\%$$

#### Total observed error

TE_obs_ was calculated from intra-assay CV and percent bias (TE_obs_ = bias% +  [2 × CV%]) for the comparisons POC1/TIA-Hum2, POC1/TIA-Vet, and TIA-Hum2/TIA-Vet. Given the absence of veterinary quality requirements for SAA, TE_obs_ was compared to the total allowable error (TE_a_) of 37% established for human SAA based on biologic variation.^6^

### Intra-method comparison

#### Interferences

To investigate possible interferences, aliquots of a pooled equine serum sample with a mean SAA concentration of ~1,650 mg/L, as assessed with the TIA-Hum test, were used. For assessment of the effect of bilirubinemia, 20 mg of bilirubin (bilirubin ≥ 98% powder; MilliporeSigma) was dissolved in 1 mL of 0.1 M NaOH to obtain a stock solution of 20 g/L. Subsequently, 0.01 mL of the product was added to 0.24 mL of a non-spiked serum sample to achieve a bilirubin concentration of 0.8 g/L. To assess the effect of hemoglobinemia, a stock solution containing 100 g/L of hemoglobin (hemoglobin from bovine blood, lyophilized powder; MilliporeSigma) was prepared by diluting 30 mg of lyophilized bovine hemoglobin in 0.3 mL of 0.9% NaCl. Then, 0.01 mL of the solution was added to 0.24 mL of non-spiked serum sample resulting in a hemoglobin concentration of 4 g/L. To investigate a possible interference of lipemia on results, 0.01 mL of intralipid (Lipovenoes 20%; Fresenius Kabi) was added to 0.24 mL of serum sample to obtain a concentration of soybean oil of 8 g/L.

As reported previously,^12^ control samples were spiked with the same volume and carrier substance as the interferent to avoid a matrix effect resulting from different sample material. The spiked samples were analyzed in comparison to control samples, namely serum aliquots spiked with equal volumes of either 100 mM NaOH (bilirubin), 0.9% NaCl (hemoglobin), or pure double-distilled water (intralipid). All spiked sera and control samples were measured in triplicate with the POC and TIA-Vet tests and then analyzed statistically.

Percent bias between the mean of the test and the control samples was used to calculate the percent observed interference effect (d_obs_%):

$$d_{\mathrm{obs}}\%=\frac{{\mathrm{mean}}_{\mathrm{test}}-{\mathrm{mean}}_{\mathrm{control}}}{{\mathrm{mean}}_{\mathrm{control}}}\times 100\%$$

The d_obs_% between test and control sample was considered acceptable if it was within ±10%, as described for equine SAA assays.^18^

#### Linearity

Linearity was evaluated for the POC, TIA-Hum, and TIA-Vet assays by manual stepwise dilution of equine serum samples with increased SAA concentrations based on initial TIA-Hum results of SAA_Low_ ~190 mg/L and SAA_High_ ~15,300 mg/L for POC measurements, of SAA_Low_ ~190 mg/L and SAA_High_ ~1,600 mg/L for TIA-Hum measurements, and of SAA_Low_ ~180 mg/L and SAA_High_ ~1,900 mg/L for TIA-Vet measurements. We performed 2 experiments to assess linearity. First, we assessed linearity within the lower concentration range covering the reportable range of each assay; second, we evaluated linearity within the high concentration range to investigate if linearity is present at high SAA concentrations, for which dilution of an equine SAA sample would be required. Additionally, we chose 2 experiments to minimize dilution error in long dilution series. To assess POC SAA_High_ linearity, the manufacturer’s dilution protocol for SAA samples with a concentration >3,000 mg/L was used. To assess linearity of all 3 assays, serial dilution resulted in specimens with 1, 0.8, 0.6, 0.4, 0.2, and 0 of the original SAA concentration using the POC manufacturer’s buffer solution for investigation of POC linearity, and 0.9% NaCl for linearity assessment of TIA-Hum and TIA-Vet tests. All diluted aliquots were analyzed in triplicate.

#### Recovery

Linearity under dilution was investigated by visual inspection of the correlation of observed SAA values plotted against a calculated (expected) SAA concentration. Percent recovery rate (RR) was calculated using the difference between actual and theoretical SAA concentration, as described for canine C-reactive protein (CRP)^14^:

$$\mathrm{RR}\left[\%\right]=\frac{\mathrm{measured}\;\;\mathrm{concentration}}{\mathrm{expected}\;\;\;\mathrm{concentration}}\times 100$$

Linear regression analysis was used to assess correlation between expected and measured results. The range of 80–120% was set as a quality goal for recovery after dilution, as described previously for immunoassay validation.^1,21^

#### Hook effect

To investigate a possible hook (prozone) effect for the POC assay, a serum sample with a remarkably high SAA concentration of ~15,300 mg/L based on TIA-Hum analysis after dilution was used. In case of a hook effect, stepwise dilution of the sample was performed to assess the SAA concentration at which a correct detection of high values without a hook effect was possible. Potential dilution steps were 1, 0.8, 0.6, 0.4, 0.2, 0.1, and 0.05 of the original SAA concentration. Each dilution step was measured in triplicate.

The presence of a hook effect was investigated by visual inspection of the observed SAA values as a function of the calculated (expected) SAA concentration. Linear regression analysis was used to assess correlation between expected and measured results. Percent RR over all concentration steps was calculated as described above, and the range of 80–120% was set as the quality goal.^1,21^

#### Storage

Given that the duration of storage ranged from 0.3 y at the beginning of the study in March 2018 to 7.3 y at the end of the study in January 2021, the impact of a variable length of storage at −80°C as well as of 2 freeze–thaw cycles on equine serum SAA were investigated at the time of TIA-Vet measurements. The same 10 equine serum samples were re-analyzed a second time with the POC method in January 2021 (subsequently POC2) and with the TIA-Hum a third time (subsequently TIA-Hum3) and compared to previous (TIA-Hum1, TIA-Hum2, and POC1) results. To assess the impact of the variable length of storage on the equine serum SAA samples, POC1, POC2, TIA-Hum1, TIA-Hum2, and TIA-Hum3 results were used. The same 10 equine SAA sera were compared at each time. A Wilcoxon signed-rank test was used to assess differences between POC1 and POC2 measurements. A nonparametric Friedmann test and a Dunn multiple comparison post-test were used for comparison of TIA-Hum1, TIA-Hum2, and TIA-Hum3 results.

## Results

### Inter-method comparison

**Figure 1.**
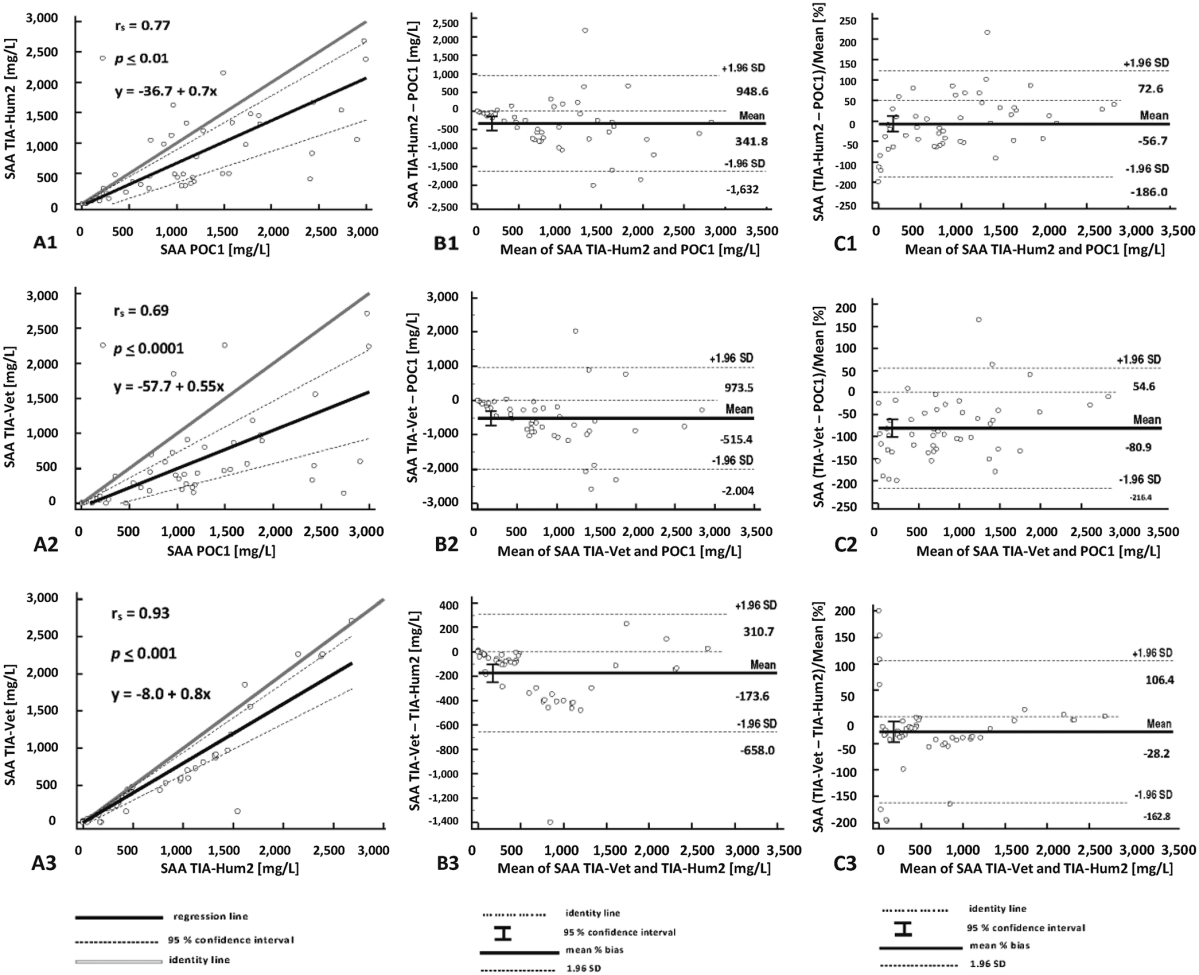
Method comparison. The point-of-care analyzer was compared to 2 turbidimetric immunoassays run on an automated large bench top analyzer (Pentra 400; ABX Horiba). **A1–3.** Passing–Bablok regression analysis with 95% CIs of serum amyloid A (SAA) measurements performed with the 3 tests. **B1–3.** Bland–Altman difference plot demonstrating the absolute bias between SAA measurements obtained with the 3 assays. **C1–3.** Bland–Altman difference plot demonstrating mean percent bias_obs_ with its 95% CI and its 1.96-fold SD indicative of its limits of agreement. Sample stability proven to be >2.5 y. POC1 = point-of-care assay, measured in 2018 (Stablelab; Zoetis); TIA-Hum2 = turbidimetric immunoassay-human, measured in 2018 (LZ-SAA assay; Eiken); TIA-Vet = turbidimetric immunoassay-veterinary (VET-SAA assay; Eiken).

There was a significant difference between median SAA measurements obtained with all analyzers (*p* < 0.0001). Highest SAA results were obtained with the POC method; lowest SAA results were analyzed with the TIA-Vet assay. Median (minimum to maximum) values obtained were 1,093 mg/L (4–3,000 mg/L), 752 mg/L (0–2,682 mg/L), and 578 mg/L (0.5–2,255 mg/L) for the POC, TIA-Hum2, and TIA-Vet assays, respectively. The dataset used for method comparison covered the complete range of interest with homogeneous data distribution. The correlation between the POC and TIA-Hum assays (Fig. 1A1–C1) as well as between the POC and TIA-Vet assays (Fig. 1A2–C2) was fair, with r_s_ = 0.77 and 0.69, respectively. Furthermore, Passing–Bablok regression analysis revealed both constant and proportional bias for the comparison of the POC and TIA-Hum assays as well as between the POC and TIA-Vet assays (Fig. 1A1, 1A2). Contrary to this bias, correlation between SAA measurements obtained with TIA-Hum and TIA-Vet tests was excellent, with r_s_ = 0.93 and only a small constant and proportional bias. All 3 assays had a growing absolute bias with increasing SAA concentration (Fig. 1B1–B3). Bland–Altman analysis revealed a proportional bias of −56.7% and −80.9% between SAA measurements obtained with the POC assay and TIA-Hum and TIA-Vet assays, respectively (Fig. 1C1, 1C2). A bias of −28.2% was observed when both TIAs were compared (Fig. 1C3). Percent bias was highest when POC and TIA-Vet tests were compared (Fig. 1A2–C2), and lowest between both TIAs (Fig. 1A3–C3).

#### Precision

POC intra- and inter-assay CVs mainly exceeded quality requirements for human SAA, except for the intra-assay CV within the medium SAA concentration of 301–1,000 mg/L. TIA-Hum and TIA-Vet assays mainly fulfilled quality requirements (CV_a_ ****<** **12.5%) for all 3 concentrations; the only exception was the TIA-Hum inter-assay CV of the SAA_High_ measurement. A broad range of intra-assay CVs was observed for the TIA-Hum test, which spanned from 3.1% in group SAA_Med_ to 40.2% in group SAA_High_ (Table 1).

**Table 1.** Intra- and inter-assay CVs obtained from replicate serum amyloid A (SAA) measurements with POC, TIA-Hum, and TIA-Vet assays.

SAA concentration range	Intra-assay CV			Inter-assay CV		
	Mean (mg/L)	SD (mg/L)	CV (%)	Mean (mg/L)	SD (mg/L)	CV (%)
POC						
0–300 mg/L	118	19	16*	86	19	22*
301–1,000 mg/L	740	56	8	249	49	20*
1,001–3,000 mg/L	1,532	460	30*	1,534	550	36*
TIA-Hum						
0–300 mg/L	34	1	5	74	6	9
301–1,000 mg/L	332	13	4	235	7	3
1,001–3,000 mg/L	1,073	17	2	2,136	860	40*
TIA-Vet						
0–300 mg/L	94	3	3	42	2	5
301–1,000 mg/L	560	14	2	127	11	8
1,001–3,000 mg/L	865	31	3	1,206	140	12

POC = point-of-care assay (Stablelab; Zoetis); TIA-Hum = turbidimetric immunoassay-human (LZ-SAA assay; Eiken); TIA-Vet = turbidimetric immunoassay-veterinary (VET-SAA assay; Eiken).

* CVs exceeding the published quality requirements for human SAA of 12.5%.

#### Total observed error

TE_obs_ of the assay comparisons POC1 versus TIA-Hum2 and POC1 versus TIA-Vet exceeded the TE_a_ of 37% established for human SAA assays.^6^ TE_obs_ was highest for the comparison of POC1 versus TIA-Vet for all investigated concentration ranges. The lowest TE_obs_ were observed for the comparison TIA-Hum2 versus TIA-Vet, which met the quality requirements of <37% (Table 2).

**Table 2.** Total observed error (%) for comparison of the assays POC, TIA-Hum, and TIA-Vet in the 3 investigated concentration ranges.

TE_obs_	TIA-Hum2 vs. POC1	TIA-Vet vs. POC1	TIA-Hum2 vs. TIA-Vet
SAA_Low_	66.3*	87.5*	34.8
SAA_Med_	64.3*	85.9*	33.2
SAA_High_	59.9*	87.9*	35.2

POC1 = point-of-care assay, measured in 2018 (Stablelab; Zoetis); SAA_High_ = group of high serum amyloid A (SAA) concentrations; SAA_Low_ = group of low SAA concentrations; SAA_Med_ = group of intermediate SAA concentrations; TE_obs_ = total observed error; TIA-Hum2 = turbidimetric immunoassay-human, measured in 2018 (LZ-SAA assay; Eiken); TIA-Vet = turbidimetric immunoassay-veterinary (VET-SAA assay; Eiken).

* TE_obs_ exceeding the published TE_a_ for human SAA of 37%.^6^

### Intra-method comparison

#### Interferences

The addition of 0.8 g/L bilirubin, 4 g/L hemoglobin, and 8 g/L intralipid resulted in detectable interferences on POC and TIA-Vet SAA results. The percent bias_obs_ exceeded the published criteria of acceptability of ±10%^18^ in all cases. Hemoglobin and bilirubin had a negative bias on SAA results measured by both POC and TIA-Vet assays; intralipid had a positive bias on SAA in both cases. The strongest bias_obs_ of 77.4% was seen when a serum sample spiked with intralipid was measured with the POC assay. A bias close to the limit of acceptance was detected for the TIA-Vet assay under hemoglobin influence (bias_obs_ = –10.6%; Table 3).

**Table 3.** Observed interference effects of bilirubin, hemoglobin, and intralipid on triplicate serum amyloid A (SAA) measurement with the POC and TIA-Vet assays.

	POC			TIA-Vet		
Interferent	Mean SAA_control_ (mg/L ± SD)	Mean SAA_test_ (mg/L ± SD)	Bias_obs_ (%)	Mean SAA_control_ (mg/L ± SD)	Mean SAA_test_ (mg/L ± SD)	Bias_obs_ (%)
Bilirubin, 0.8 g/L	1,321 ± 504	1,106 ± 100	−16*	959 ± 55	710 ± 18	−26*
Hemoglobin, 4 g/L	2,224 ± 940	1,211 ± 281	−46*	730 ± 10	653 ± 12	−11*
Intralipid, 8 g/L	1,054 ± 179	1,870 ± 404	77*	768 ± 18	1,043 ± 65	36*

POC = point-of-care assay (Stablelab; Zoetis); TIA-Vet = turbidimetric immunoassay-veterinary (VET-SAA assay; Eiken).

* Results of the observed bias for the interfering substance exceeding 10%.^18^

#### Linearity

**Figure 2.**
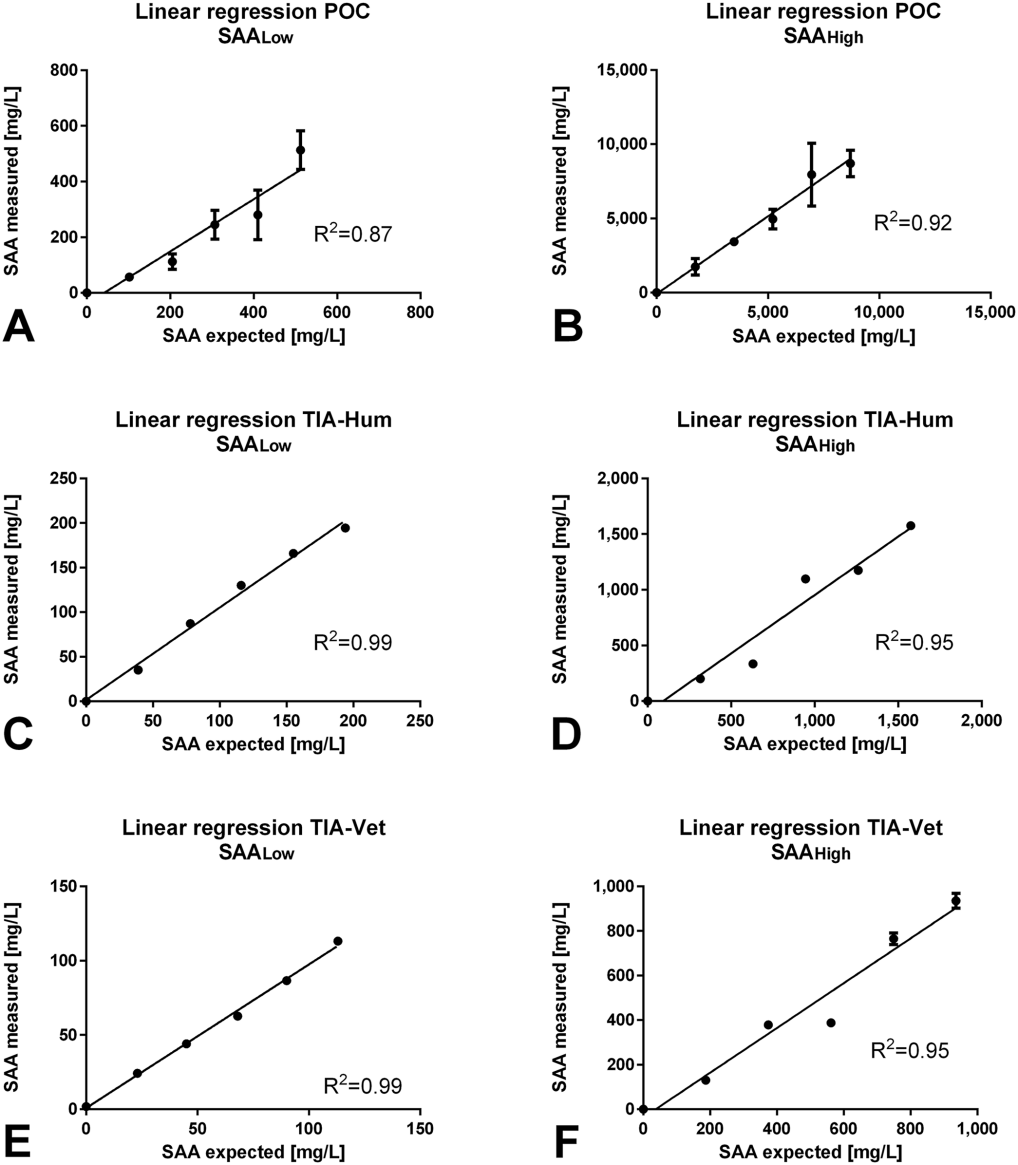
Linearity of diluted equine serum samples measured with the POC, TIA-Hum, and TIA-Vet assays for **A.** POC measurement of a sample originally containing 190 mg/L serum amyloid A (SAA), **B.** POC measurement of a sample originally containing 15,300 mg/L SAA, **C.** TIA-Hum measurement of a sample originally containing 190 mg/L SAA, **D.** TIA-Hum measurement of a sample originally containing 1,600 mg/L SAA, **E.** TIA-Vet measurement of a sample originally containing 180 mg/L SAA, and **F.** TIA-Vet measurement of a sample originally containing 1,900 mg/L SAA. A serial dilution was performed in all cases to achieve 6 different SAA concentrations (i.e., 1, 0.8, 0.6, 0.4, 0.2, 0 parts of the original concentration). Original SAA concentrations were assessed with the TIA-Hum method. POC = point-of-care assay (Stablelab; Zoetis); TIA-Hum = turbidimetric immunoassay-human (LZ-SAA assay; Eiken); TIA-Vet = turbidimetric immunoassay-veterinary (VET-SAA assay; Eiken).

The POC test was linear (*R*^2^ = 0.87) when SAA_Low_ samples of 0–562 mg/L were analyzed (Table 4; Fig. 2A). POC linearity was superior (*R*^2^ = 0.92) when SAA_High_ concentrations of 0–10,312 mg/L were measured (Table 4; Fig. 2B). POC SAA results were highly variable within SAA_Low_ and SAA_High_ triplicates (Table 4; Figs. 2A, 2B). For example, POC results were 4,904–10,312 mg/L when the concentration of 0.8 was measured (Fig. 2B). Both the TIA-Hum and TIA-Vet tests had excellent linearity (*R*^2^ = 0.99 each) when SAA_Low_ samples of 0–198 mg/L and 1–115 mg/L, respectively, were measured (Table 4; Figs. 2C, 2E). TIA-Hum and TIA-Vet tests had good linearity (*R*^2^ = 0.95 each) for measurement of SAA_High_ samples of 0–1,593 mg/L (TIA-Hum) and 0–969 mg/L (TIA-Vet; Table 4, Fig. 2D, 2F). When the SAA_High_ sample was measured with the TIA-Vet test and the dilution factors 0.4 and 0.6 were applied, no relevant difference in mean SAA results of 379 and 388 mg/L, respectively, was noted (Table 4; Fig. 2F).

**Table 4.** Linearity and recovery rates of serum amyloid A (SAA) measurements performed with equine serum samples originally containing 190 mg/L (SAA_Low_/POC+TIA-Hum), 180 mg/L (SAA_Low_/TIA-Vet), 15,300 mg/L (SAA_High_/POC), 1,600 mg/L (SAA_High_/TIA-Hum), and 1,900 mg/L (SAA_High_/TIA-Vet) SAA as determined by the TIA-Hum method.

DS	SAA_Low_									SAA_High_								
	POC			TIA-Hum			TIA-Vet			POC			TIA-Hum			TIA-Vet		
	RR (%)	Mean_exp._ (mg/L)	Mean_meas._ (mg/L)	RR (%)	Mean_exp._ (mg/L)	Mean_meas._ (mg/L)	RR (%)	Mean_exp._ (mg/L)	Mean_meas._ (mg/L)	RR (%)	Mean_exp._ (mg/L)	Mean_meas._ (mg/L)	RR (%)	Mean_exp._ (mg/L)	Mean_meas._ (mg/L)	RR (%)	Mean_exp._ (mg/L)	Mean_meas._ (mg/L)
1	100	512	512	100	194	194	100	113	113	100	8,706	8,706	100	1,575	1,575	100	936	936
0.8	68*	281	410	106	155	166	96	90	87	78*	6,965	7,960	93	1,260	1,174	102	749	765
0.6	79*	245	307	111	116	130	93	68	63	110	5,223	4,953	116	945	1,097	69*	562	388
0.4	55*	113	205	111	78	87	97	45	44	65*	3,482	3,433	53*	630	334	101	374	379
0.2	56*	57	102	89	39	35	107	23	24	47*	1,741	1,746	63*	315	200	69*	187	131
0	100	0	0	100	0	0	100	0	2	100	0	0	100	0	0	100	0	0.8

DS = dilution step; mean_exp._ = mean expected (calculated) SAA concentration; mean_meas._ = mean measured SAA concentration; POC = point-of-care assay (Stablelab; Zoetis); RR = recovery rate; SAA_High_ = group of high SAA concentrations; SAA_Low_ = group of low SAA concentrations; TIA-Hum = turbidimetric immunoassay-human (LZ-SAA assay; Eiken); TIA-Vet = turbidimetric immunoassay-veterinary (VET-SAA assay; Eiken).

* RRs below the published quality criteria for immunoassays of 80–120%.^1,10^

#### Recovery

RR did not fulfill the quality criteria of 80–120% reported for immunoassays,^1,21^ especially regarding POC assay performance in both SAA_Low_ and SAA_High_ concentration ranges and almost every dilution step (Table 4). The RR of the TIA-Hum assay fulfilled quality criteria in almost all dilution steps of SAA_Low_ and SAA_High_ measurements, except when the dilutions 0.2 and 0.4 of the original SAA_Low_ concentration were measured. The TIA-Vet test performed below the expected RR quality goals when dilution steps 0.2 and 0.6 of the SAA_High_ sample were analyzed.

#### Hook effect

**Figure 3.**
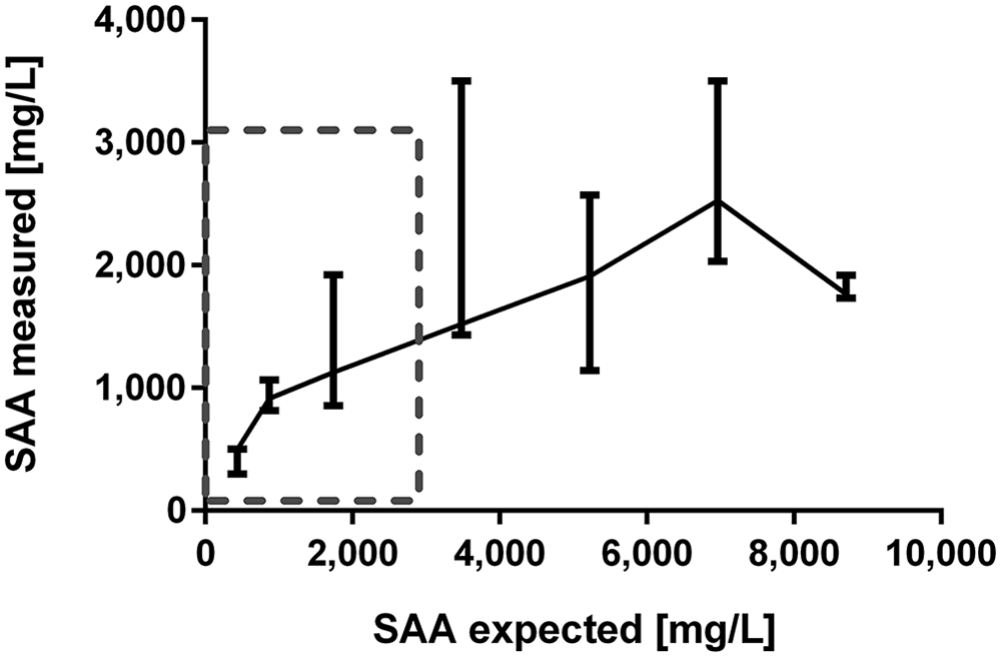
A hook effect was present in POC (point-of-care; Stablelab, Zoetis) assay measurements when serum amyloid A (SAA) values >3,000 mg/L exceeded the original working range of the assay. Linearity under dilution of an equine serum sample originally containing 15,300 mg/L SAA as determined by the TIA-Hum (turbidimetric immunoassay-human; LZ-SAA assay, Eiken) method is shown. The gray box marks the original concentration range of the assay. SAA values reported by the POC assay as “>3,000 mg/L” are replaced with 3,500 mg/L. A serial dilution was performed to achieve 7 different SAA concentrations (i.e., 1, 0.8, 0.6, 0.4, 0.2, 0.1, 0.05 parts of the original concentration).

A hook effect was observed at SAA concentrations >3,000 mg/L, which exceeded the original working range of the POC assay. A hook effect occurred from dilution step 0.4 of the original SAA concentration (Fig. 3). There was extensive variation of SAA results (minimum to maximum) within triplicate measurements of the dilution steps 0.2 (855–1,921 mg/L), 0.4 (1,433 to >3,000 mg/L), 0.6 (1,143–2,573 mg/L), and 0.8 (2,033 to >3,000 mg/L) of the original SAA concentration. The POC assay correctly reported SAA values of “>3,000 mg/L” only in 2 of 12 measurements, where expected SAA concentrations exceeded 3,000 mg/L. Total RR calculated over all dilution steps was 39%, which was significantly below the quality goal for immunoassays of 80–120%.^1,21^

#### Storage

**Figure 4.**
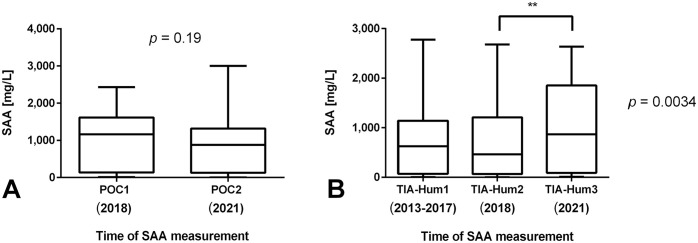
Impact of storage on equine serum amyloid A (SAA) samples demonstrated with POC assay results and TIA-Hum results obtained at different times after storage. TIA-Hum1 represents the original measurement during routine testing in 2013–2017; TIA-Hum2 and POC1 the measurement in 2018 after a median storage of 2.5 y (range: 0.3–4.8 y); TIA-Hum3 and POC2 results were obtained January 2021 (i.e., a median of 2.5 y after the previous measurement). Box-and-whisker diagram demonstrates median range of the SAA measurements obtained with the 2 analyzers at different times. The horizontal line in the boxes is the median, the whiskers indicate the range, and the box represents the 25th–75th percentile. The asterisks indicate the strength of the *p*-value; **p ≤ 0.01. **A.** TIA-Hum results at 3 different times (1–3) were compared. **B.** POC results at 2 different times (1–2) were compared. POC1–POC2 = point-of-care assay, measured in 2018 and 2021, respectively (Stablelab; Zoetis); TIA-Hum1–TIA-Hum3 = turbidimetric immunoassay-human (LZ-SAA assay; Eiken), measured in 2013–2017, 2018, and 2021, respectively.

The impact of storage on equine serum SAA results was measured with the POC and TIA-Hum tests. Median (minimum to maximum) SAA results obtained were 1,080 mg/L (11–2,430 mg/L) and 925 mg/L (0–3,000 mg/L) when POC1 and POC2 results were compared. As such, no significant impact of storage was observed (*p* = 0.19; Fig. 4A). For TIA-Hum1, TIA-Hum2, and TIA-Hum3 measurements, median (minimum to maximum) values obtained were 778 mg/L (5–2,775 mg/L), 744 mg/L (1–2,682 mg/L), and 975 mg/L (9–2,637 mg/L), respectively. Although there was no significant difference between TIA-Hum1 and TIA-Hum2 values, TIA-Hum2 results were significantly lower than TIA-Hum3 results (*p* = 0.0034; Fig. 4B).

## Discussion

POC, TIA-Hum, and TIA-Vet results cannot be used interchangeably because of significant proportional bias_obs_ of −56.7, −80.9, and −28.8% for comparison of POC/TIA-Hum, POC/TIA-Vet, and TIA-Hum/TIA-Vet results, respectively, with general overestimation of POC results compared to the reference method. Biases between different test methods and overestimation of POC results in comparison to TIA results have been described: an overestimation of Stablelab POC results in comparison to TIA-Hum results was reported,^29^ and a proportional bias_obs_ of 19.7% was detected when POC and TIA methods for canine CRP measurement were compared.^14^

Several factors contribute to biases between different assay types, such as user-dependent errors (dilution and pipetting errors),^14^ inter-batch imprecision,^29^ use of different analyzers,^13,14,19^ and “hardware errors” caused by biochemical assay background reactions.^14^ Given that trained personnel performed all measurements in our study, user-dependent errors were minimized.^14^ The Pentra 400 analyzer was used for all TIA measurements to exclude performance variations caused by the use of different analyzers. Furthermore, variation of antibody cross-reactivity can be observed between different assay types^17^ and between different batches of the same assay,^24^ which is a known problem in immunologic acute-phase protein analysis.^9,29^ POC, TIA-Hum, and TIA-Vet assays use heterologous anti-human SAA1 antibody to detect equine SAA.^17,18,29^ However, human TIA antibody cross-reactivity might vary^24^ or even be insufficient for detection of animal blood analytes, as shown for measurement of canine CRP with a TIA based on anti-human CRP antibody.^9^ Additionally, heterologous calibration of the TIAs based on recombinant human SAA results in expression of equine SAA concentrations in human equivalents and hence different concentration read-outs.^4,18^ Affinity of anti-human SAA antibody toward equine SAA might vary between different assays,^17^ with higher affinity translating into a higher SAA concentration read from the calibration curve.^18^

Our results call for the awareness of clinical pathologists and practitioners working with heterologous immunoassays. Even long-established assays may develop variances in cross-reactivity^15^ and in test performance and reliability, which is a well-known problem in veterinary laboratories.^3^ Especially in assays with known species-dependent batch-to-batch variances, brief validations of every assay batch are recommended.^3^ In assays generally known to give reliable results, as in our case, the laboratory may take assay validation studies including 20–40 samples into account if implausible results occur after a new batch has been obtained. Future research should be aimed at improvement of species-specific assays, as in our study.

Both TIA tests had excellent agreement (r_s_ = 0.93), but the TIA-Vet method yielded 28.2% lower results compared to the TIA-Hum test in the method comparison study (*p* ≤ 0.01). Even though TIA-Vet is designed for measurement of especially high SAA concentrations, 8% lower SAA results have been described for this method when compared to the TIA-Hum method.^18^ These findings can be the result of inter-batch and antibody cross-reactivity variations.^17,18^ Hence, follow-up examinations of the patient should be made based on the same SAA assay type. Furthermore, the narrower reported working range of the TIA-Vet test (5–200 mg/L) compared to the TIA-Hum assay (5–500 mg/L) might be explained by the fact that the TIA-Vet assay exclusively uses monoclonal rat anti-human SAA1 antibody, whereas the TIA-Hum test is based on a combination of monoclonal rat anti-human SAA1 antibody and polyclonal rabbit anti-human SAA1 antibody.^17,18^

Although POC intra-assay CVs were mainly >12.5%, except in the concentration group SAA_Med_ (7.6%), TIA-Hum and TIA-Vet intra-assay CVs (1.6–4.8% and 2.5–3.5%, respectively) fulfilled the quality specifications of CV_a_ ****<** **12.5% when compared to CV_i_ described for human SAA.^21^ Intra-assay CVs of 13–18% were published for the Stablelab POC assay for SAA concentrations, consistent with the low and medium concentrations of our study, and are comparable to the CVs that we obtained for the same SAA concentration range.^29^ We observed a markedly higher intra-assay CV of 30% for SAA concentrations in the high concentration range of the POC; however, a comparably high concentration was not assessed in the previous study.^29^

Previously obtained TIA-Vet intra-assay CVs of 3–5.2%^18^ are in agreement with our results and confirm its good intra-assay repeatability in our study.

In agreement with our findings, a high inter-assay CV of up to 45.5% was reported previously for the POC assay and was considered to be the result of the use of different sample types (serum and plasma) and batch-to-batch differences.^29^ Impact of sample type and of batch, however, was excluded in our study because we used only serum samples and test cartridges of the same batch.

For high SAA concentrations (>500 mg/L), the TIA-Hum inter-assay CV of 40.2% markedly exceeded suggested quality requirements for human SAA. When SAA concentrations exceeded 500 mg/L, automatic dilution steps were performed by the analyzer during each analysis. Thus, errors caused by additional dilution steps contribute to imprecision and are considered the most likely reason for the high inter-assay CV observed for high SAA concentrations that exceed the original working range of the test.

In contrast to the POC, TIA-Vet inter-assay CVs were <12.5% and fulfilled human SAA quality requirements for measurement of high SAA concentrations, which is in agreement with the previous study.^18^

Only TE_obs_ of the comparison of TIA-Hum2 versus TIA-Vet was less than the TE_a_ of 37% based on biologic variation established for human SAA assays.^6^ To date, there are no published data on analytical quality requirements for equine SAA,^29^ hence comparison to TE_a_ for human SAA, as well as comparison of CV_a_ to human CV_i_, can only serve as a rough guideline.

The addition of the interferents hemoglobin, bilirubin, and intralipid resulted in a bias on POC and TIA-Vet results exceeding the accepted deviation of ±10%.^18^ Investigation of the impact of interferences was warranted given that the POC methodology relies on a color change, and conditions such as hemolysis, hyperbilirubinemia, and lipemia can affect results.^29^ Although there was no interference effect on TIA-Vet results under the influence of 10 g/L hemoglobin and 5 g/L intralipid in a previous study,^18^ the addition of 4 g/L hemoglobin, 8 g/L intralipid, and 0.8 g/L bilirubin had a positive percent bias_obs_ on POC and TIA-Vet results in the case of intralipid, and a negative percent bias_obs_ in the cases of hemoglobin and bilirubin. The different results of our study compared to the reference study may be explained by different spike protocols used by the researchers. Impact of the interferents hemoglobin, bilirubin, and intralipid on equine serum SAA results must be considered when analyzing samples of horses with relevant clinical conditions.

POC linearity was good (*R*^2^ = 0.92) from 0–10,312 mg/L, and inferior (*R*^2^ = 0.87) from 0–562 mg/L, with fair agreement between measured and expected results. This is in contrast to a previous publication that describes an acceptable POC linearity up to 1,000 mg/L and less precise results >1,000 mg/L, with POC overestimation of samples >3,000 mg/L compared to TIA-Hum (Eiken LZ-SAA) results.^29^ The dilution protocol provided by the manufacturer of the POC was considered inadequate to obtain linear results by the authors of the previous method validation study.^29^ In contrast, the manufacturer’s dilution protocol for the POC led to good linearity up to the highest measured SAA concentrations in our study. The manufacturer’s dilution protocols might have been revised since publication of the previous study.

TIA-Hum and TIA-Vet assays were linear up to SAA concentrations of 1,593 and 969 mg/L, respectively, with overall good agreement of measured and expected results. Minor signs of inaccuracy consisted of slight underestimation of TIA-Hum and TIA-Vet results in the SAA_High_ group. A possible explanation for this observed underestimation is the heterologous calibration of SAA assays reporting equine SAA results as human equivalents.^18^

When the SAA_High_ sample was measured with the TIA-Vet test in dilution steps 0.4 and 0.6, respectively, no difference between expected and measured mean SAA results was noted despite the different degree of dilution. The fact that no difference between expected and measured mean SAA results was noted could be explained by a dilution error during preparation of dilution step 0.6 given that all other results closely met the expected values. Clustering of original TIA-Hum results of ~400 mg/L was observed in a previous study when dilutions recommended for human SAA measurement were applied^29^; a 1:6 dilution was suggested for TIA-Hum SAA measurements >250 mg/L.^17,29^ In accordance with the previous recommendation, clustering of SAA values was not observed for both TIA-Hum and TIA-Vet in our study in which an automated TIA-Hum 1:6 dilution was made for SAA exceeding the upper measuring range of the test, suggesting appropriate dilution protocols for measurement of equine SAA on the Pentra 400.

RR for the POC, TIA-Hum, and TIA-Vet tests were 47–110%, 53–116%, and 69–107%, respectively, and therefore partially diverged from the quality goals of 80–120% published for immunoassays.^1,21^ Low RR occurred especially within the higher concentration range of the linearity experiment; pipetting errors could have been introduced by the need for extensive dilution.^10^

A hook effect was observed at SAA concentrations >3,000 mg/L, exceeding the original working range of the POC assay. This finding is in accordance with the manufacturer’s information. SAA concentrations >3,000 mg/L are frequently encountered in horses.^18,33^ Relevant changes at very high SAA concentrations cannot be tracked reliably with the present POC assay. However, good linearity (*R*^2^ = 0.92) was achieved for the same SAA_High_ sample when the manufacturer’s dilution protocol was applied in the linearity study. Based on our results, it is advisable to perform repeat SAA measurements using the manufacturer’s dilution protocol when clinical appearance of a horse and SAA results do not correlate and hence may represent falsely low concentrations secondary to a possible hook effect.

A major limitation of our study is that it was conducted over the course of several years, hence making 2 freeze–thaw cycles necessary to obtain separate aliquots. However, the median sample age difference of 2.5 y had no impact on equine serum SAA stability in our study. Although previous studies show that equine serum SAA is stable over 17 d when stored at room temperature and refrigerated at 4°C,^11^ our study suggests that equine serum SAA is stable for much longer when stored at −80°C. TIA-Hum3 results that were obtained after a median of 2.5 y after TIA-Hum2 results were significantly higher than TIA-Hum2 results (*p* ≤ 0.01). This is in contrast to other studies that report a general decrease of stored blood analytes.^25,32^ Furthermore, it has been reported that samples may be degraded by repeated freeze–thaw cycles.^2^ Given that different TIA-Hum batches were used for TIA-Hum2 and TIA-Hum3 measurements, it is most likely that the spurious SAA increase after storage and repeated freeze–thaw cycles was the result of an inter-batch imprecision with different antibody cross-reactivity of TIA-Hum anti-human SAA antibody to equine SAA, as reported previously.^17,18^

## References

[1] Andreasson U , et al. A practical guide to immunoassay method validation. Front Neurol 2015;6:179. 26347708 10.3389/fneur.2015.00179 PMC4541289

[2] Arnold JE , et al. ASVCP guidelines: principles of quality assurance and standards for veterinary clinical pathology (version 3.0): developed by the American Society for Veterinary Clinical Pathology’s (ASVCP) Quality Assurance and Laboratory Standards (QALS) Committee. Vet Clin Pathol 2019;48:542–618. 31889337 10.1111/vcp.12810

[3] Cerón JJ , et al. A seven-point plan for acute phase protein interpretation in companion animals. Vet J 2008;177:6–7. 18294886 10.1016/j.tvjl.2007.12.001

[4] Christensen M , et al. Evaluation of an automated assay based on monoclonal anti-human serum amyloid A (SAA) antibodies for measurement of canine, feline, and equine SAA. Vet J 2012;194:332–337. 22704135 10.1016/j.tvjl.2012.05.007

[5] Coutinho da Silva MA , et al. Serum amyloid A concentration in healthy periparturient mares and mares with ascending placentitis. Equine Vet J 2013;45:619–624. 23432049 10.1111/evj.12034

[6] d’Eril GM , et al. Biological variation of serum amyloid a in healthy subjects. Clin Chem 2001;47:1498–1499. 11468251

[7] Eckersall PD Bell R . Acute phase proteins: biomarkers of infection and inflammation in veterinary medicine. Vet J 2010;185:23–27. 20621712 10.1016/j.tvjl.2010.04.009

[8] Flatland B , et al. ASVCP guidelines: quality assurance for point-of-care testing in veterinary medicine. Vet Clin Pathol 2013;42:405–423. 24320778 10.1111/vcp.12099

[9] Fransson BA , et al. Assessment of three automated assays for C-reactive protein determination in dogs. Am J Vet Res 2007;68:1281–1286. 18052731 10.2460/ajvr.68.12.1281

[10] Harr KE , et al. ASVCP guidelines: allowable total error guidelines for biochemistry. Vet Clin Pathol 2013;42:424–436. 24320779 10.1111/vcp.12101

[11] Hillström A , et al. Evaluation of an in-clinic serum amyloid A (SAA) assay and assessment of the effects of storage on SAA samples. Acta Vet Scand 2010;52:8. 20122257 10.1186/1751-0147-52-8 PMC2831015

[12] Hillström A , et al. Validation and application of a canine-specific automated high-sensitivity C-reactive protein assay. J Vet Diagn Invest 2015;27:182–190. 25776543 10.1177/1040638715575751

[13] Hindenberg S , et al. Evaluation of a species-specific C-reactive protein assay for the dog on the ABX Pentra 400 clinical chemistry analyzer. BMC Vet Res 2017;13:146. 28558755 10.1186/s12917-017-1065-9 PMC5450169

[14] Hindenberg S , et al. Evaluation of a novel quantitative canine species-specific point-of-care assay for C-reactive protein. BMC Vet Res 2018;14:99. 29554960 10.1186/s12917-018-1415-2 PMC5859731

[15] Ishioka K Hayakawa N . Serum amyloid A concentrations in cats measured using a newly developed feline-specific latex agglutination immunoassay. Jpn J Vet Res 2019;67:145–150.

[16] Jacobsen S Andersen PH . The acute phase protein serum amyloid A (SAA) as a marker of inflammation in horses. Equine Vet Educ 2007;19:38–46.

[17] Jacobsen S , et al. Evaluation of a commercially available human serum amyloid A (SAA) turbidometric immunoassay for determination of equine SAA concentrations. Vet J 2006;172:315–319. 15950503 10.1016/j.tvjl.2005.04.021

[18] Jacobsen S , et al. Validation of an equine serum amyloid A assay with an unusually broad working range. BMC Vet Res 2019;15:462. 31856804 10.1186/s12917-019-2211-3 PMC6923866

[19] Jasensky A-K , et al. Evaluation of three different point-of-care tests for quantitative measurement of canine C-reactive protein. Vet Clin Pathol 2015;44:205–214. 25962332 10.1111/vcp.12264

[20] Jensen AL Kjelgaard-Hansen M . Method comparison in the clinical laboratory. Vet Clin Pathol 2006;35:276–286. 16967409 10.1111/j.1939-165x.2006.tb00131.x

[21] Jensen AL Kjelgaard-Hansen M . Diagnostic test validation. In: Weiss DJ Wardrop KJ , eds. Schalm’s Veterinary Hematology. 6th ed. Wiley Blackwell, 2010:1027–1033.

[22] Karam B , et al. Whole-blood validation of a new point-of-care equine serum amyloid A assay. J Equine Vet Sci 2020;94: 103222. 33077080 10.1016/j.jevs.2020.103222

[23] Kjelgaard-Hansen M Jacobsen S . Assay validation and diagnostic applications of major acute-phase protein testing in companion animals. Clin Lab Med 2011;31:51–70. 21295722 10.1016/j.cll.2010.10.002

[24] Klenner S , et al. Evaluation of three automated human immunoturbidimetric assays for the detection of C-reactive protein in dogs. J Vet Diagn Invest 2010;22:544–552. 20622224 10.1177/104063871002200408

[25] Marjani A . Effect of storage time and temperature on serum analytes. Am J Appl Sci 2008;5:1047–1051.

[26] Nunokawa Y , et al. Evaluation of serum amyloid A protein as an acute-phase reactive protein in horses. J Vet Med Sci 1993;55:1011–1016. 7509640 10.1292/jvms.55.1011

[27] Papasouliotis K , et al. Comparison of white blood cell differential percentages determined by the in-house LaserCyte hematology analyzer and a manual method. Vet Clin Pathol 2006;35:295–302. 16967412 10.1111/j.1939-165x.2006.tb00134.x

[28] Pepys MB , et al. Serum amyloid A protein (SAA) in horses: objective measurement of the acute phase response. Equine Vet J 1989;21:106–109. 2539996 10.1111/j.2042-3306.1989.tb02108.x

[29] Schwartz D , et al. Analytical validation of a new point-of-care assay for serum amyloid A in horses. Equine Vet J 2018;50:678–683. 29344980 10.1111/evj.12807

[30] Stack JD , et al. Comparison of serum amyloid A measurements in equine synovial fluid with routine diagnostic methods to detect synovial infection in a clinical environment. Front Vet Sci 2019;6:325. 31632987 10.3389/fvets.2019.00325 PMC6779708

[31] Tape C Kisilevsky R . Apolipoprotein A-I and apolipoprotein SAA half-lives during acute inflammation and amyloidogenesis. Biochim Biophys Acta 1990;1043:295–300. 2108727 10.1016/0005-2760(90)90030-2

[32] Verma M , et al. Effect of blood storage on complete biochemistry. J Blood Disord Transfus 2015;6:329.

[33] Viner M , et al. Comparison of serum amyloid A in horses with infectious and noninfectious respiratory diseases. J Equine Vet Sci 2017;49:11–13.

[34] Westgard JO , et al. Criteria for judging precision and accuracy in method development and evaluation. Clin Chem 1974;20:825–833. 4835236

[35] Witkowska-Piłaszewicz OD , et al. Serum amyloid A in equine health and disease. Equine Vet J 2019;51:293–298. 30565319 10.1111/evj.13062 PMC7163734

